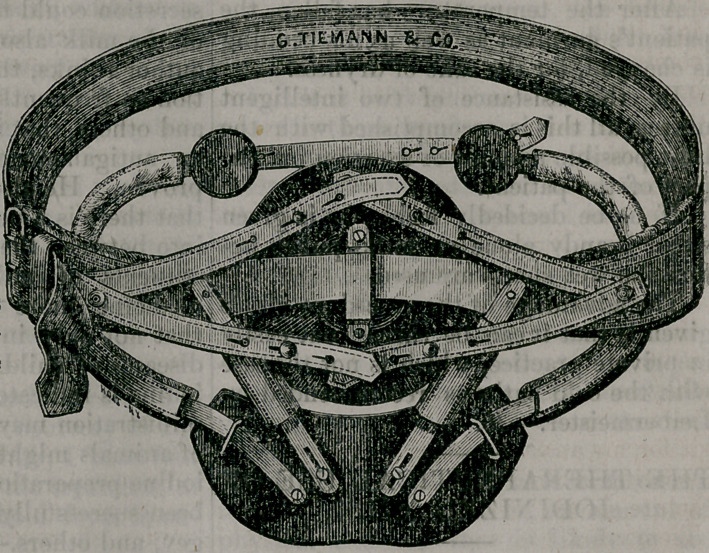# Case of Umbilical Hernia

**Published:** 1878-08-20

**Authors:** B. M. Walker

**Affiliations:** Danville, Va.


### CASE OF UMBILICAL HERNIA.

By B. M. Walker, M.D., Of Danville, Va.

I was called to see Mrs. G. in February last. I was told by her husband, who came for me, that she was suffering from an "enlargement in her abdomen; that sometimes she was better, and then, from no apparent cause, she would get rapidly worse." From his attempts at description I suspected hernia in some form.

Upon examination of the patient, I found her to be a very large, fleshy woman, weighing two hundred and twenty pounds; abdomen pendulous, and the walls well stored with adipose tissue. Protruding from the umbilical ring was a hard, strangulated mass. Certain rational symptoms clearly indicated the existence of intestine in the imprisoned substance. After careful taxis under partial anæsthesia, I found the stricture exceedingly unyielding. Having to deal with so much fat in the adjacent structures that it seriously impaired my efforts at taxis, I expressed the doubt to her husband and herself, as to the efficiency of any means of relief short of operation, which I feared I would have to perform. This she streuuously objected to, saying, "she was going to die, any way," urging me to relieve her again by anæsthesia.

Bringing my patient quite under the influence of chloroform the second time, preceded by a hopodermic injection of morphia, and applying ice and ether locally to the tumor, by prolonged and careful efforts the hernia was reduced, *i.e.*, the intestine was liberated; but I found, then, a small, adherent substance attached to one side of the ring. The patient's bowels were soon relieved, and in the course of twelve hours sufficient evidence of thorough evacuation of the alimentary canal had occurred. This satisfied me of the nature of the remaining substance of the tumor. Considerable tenderness supervened; and a fever heat to 104.5 deg. Fahr., for forty-eight hours, was treated by cool applications over the abdomen, and fluid extract of gelseminum, as circumstances demanded.

Upon the restoration to health, I found the enlargement at the ring gradually decreasing. I used iodine liniment, and kept up a soluble state of the bowels. At the expiration of two weeks the tumor had entirely disappeared, leaving a larger opening than before its removal. Through this umbilical outlet masses of intestine and omentum not unfrequently protruded, despite the efforts at restraint made by the temporary dressings and instruments used. The patient was cautioned (and I think needlessly so, for her recent sufferings were vividly before her.) She affirmed that the weight of her abdomen, when standing, would displace any appliance to the umbilical ring. All efforts to restrict the protrusion prevailed but a short time.

Seeing that an abdominal supporter was indispensable in the management of the case (an operation for radical cure being opposed as persistently as was that suggested for relief of the stricture), I found that her protection must be instrumental. I could not adjust both supporter and truss (each of which were urgently demanded) without strangulating, as it were, large masses and folds of the abdominal walls intervening between the upper border of the supporter and the lower margin of the truss. Nor would this difficulty have been obviated had I had a larger, or rather higher, supporter, as the motions of the body would have permitted the displacement of one or the other of the instruments, from the integument gradually insinuating itself between, and eventually becoming a source of pain and discomfort, or else displacing the protectors.

Under these circumstances the genius of invention was appealed to. As my memory had failed to suggest any remedy in the various instruments devised heretofore for similar situations, I drew a diagram for a combination truss and abdominal supporter, which I sent to that most excellent house of George Tiemann & Co., New York. In a few days they sent the instrument perfect in every particular. I lost no time in adjusting it for my anxious patient, who has been wearing it with infinite delight and absolute safety, for three months. Messrs. Tiemann & Co. were so delighted with the invention, as they call it, that they have had an electrotype taken of it and forwarded to me. I have sent it to you, with this article.—*Medical and Surgical Reporter*.

---

Dr. Q. C. Smith, of Cloverdale, conveys a valuable hint in the following note: "When cow's milk is found to disagree with hand-fed babies or small children, it may, in many cases, be rendered entirely wholesome to them by adding to it a small portion of table salt, just enough to be perceptible to the taste. I have for years directed the practice of this expedient among our people, and I know it to be of real value."—*Pacific Med. and Surg. Jour.*

## Figures and Tables

**Figure f1:**